# Internal Transcribed Spacer 1 Secondary Structure Analysis Reveals a Common Core throughout the Anaerobic Fungi (Neocallimastigomycota)

**DOI:** 10.1371/journal.pone.0091928

**Published:** 2014-03-24

**Authors:** Christian Koetschan, Sandra Kittelmann, Jingli Lu, Djamila Al-Halbouni, Graeme N. Jarvis, Tobias Müller, Matthias Wolf, Peter H. Janssen

**Affiliations:** 1 AgResearch Ltd., Grasslands Research Centre, Palmerston North, New Zealand; 2 University of Würzburg, Department of Bioinformatics, Biocenter, Würzburg, Germany; 3 ViaLactia Biosciences Ltd., Newmarket, Auckland, New Zealand; 4 New Zealand Trade and Enterprise, Wellington, New Zealand; The Scripps Research Institute, United States of America

## Abstract

The internal transcribed spacer (ITS) is a popular barcode marker for fungi and in particular the ITS1 has been widely used for the anaerobic fungi (phylum Neocallimastigomycota). A good number of validated reference sequences of isolates as well as a large number of environmental sequences are available in public databases. Its highly variable nature predisposes the ITS1 for low level phylogenetics; however, it complicates the establishment of reproducible alignments and the reconstruction of stable phylogenetic trees at higher taxonomic levels (genus and above). Here, we overcame these problems by proposing a common core secondary structure of the ITS1 of the anaerobic fungi employing a Hidden Markov Model-based ITS1 sequence annotation and a helix-wise folding approach. We integrated the additional structural information into phylogenetic analyses and present for the first time an automated sequence-structure-based taxonomy of the ITS1 of the anaerobic fungi. The methodology developed is transferable to the ITS1 of other fungal groups, and the robust taxonomy will facilitate and improve high-throughput anaerobic fungal community structure analysis of samples from various environments.

## Introduction

Anaerobic fungi of the phylum Neocallimastigomycota [1], [2] play a major role in the degradation of fibrous plant material in the gastro-intestinal tracts of ruminant and non-ruminant herbivores. This peculiar phylum of fungi, members of which were first observed by Orpin [3], consists of only one family (Neocallimastigaceae). To date, six genera of anaerobic fungi have been described based on morphological and molecular characteristics: *Anaeromyces*, *Caecomyces*, *Cyllamyces*, *Neocallimastix*, *Orpinomyces* and *Piromyces*. Recently, the internal transcribed spacers 1 and 2 (ITS1 and ITS2) were confirmed to be the best suited marker genes for all classes of fungi [4] and to provide complementary phylogenetic information [5]–[7]. Using these molecular tools, several new groups of anaerobic fungi have been detected [8]–[11]. With the increased rate of discovery of novel sequence types through the application of next-generation sequencing, taxonomic classification of environmental sequences as well as the curation of reference databases are understood to be the next major challenges for fungal taxonomy to enable the description and evaluation of fungal diversity in the environment [12], [13]. While ITS2 reference sequences of anaerobic fungi are scarce, a revised taxonomic framework has been proposed for the Neocallimastigomycota based on publicly available ITS1 sequences of isolated and morphologically identified, as well as potentially novel species [10]. This framework provides a basis for the assignment of anaerobic fungal ITS1 sequence data derived from molecular analysis of communities in diverse habitats. However, the high sequence variability, which makes the ITS1 such a valuable phylogenetic marker, at the same time makes reproducible alignments of sequences by different research groups almost impossible. A potential remedy is to integrate data from both primary sequence and secondary structure into the alignment as well as treeing methods. For the ITS2 of eukaryotes, a common secondary structure has been discovered [14], [15]. This finding allowed the development of simultaneous sequence-€structure-based alignment and treeing software, which has been successfully integrated into phylogenetic pipelines and analyses [16]–[20]. In contrast to earlier studies, where secondary structure was used to guide sequence alignments, species are scored based on a validated scoring matrix which takes into account both the variable primary sequence as well as the conserved secondary structure. The resulting similarity scores place species into phylogenetic relation to each other. This method allowed the use of ITS2 even for elucidating high level phylogenetic relationships [21]–[23], expressed as significantly more robust and more accurate tree reconstructions [24]. To date, little information is available on the secondary structure of the eukaryotic ITS1. However, group-specific core structures have been recognized at genus [25], family [26] and order [27], [28] levels. Here we present a sequence-structure-based analysis for anaerobic fungi based on the marker ITS1, following the pipeline originally described by Schultz and Wolf for the ITS2 [29]. The phylogenetic reconstruction of neocallimastigomycete sequences involved (i) the exact delineation of ITS1 sequences by applying a Hidden Markov Model (HMM)-based annotation that identifies the very conserved bordering regions of 18S and 5.8S rRNA genes, (ii) the identification and description of a common core secondary structure by evaluating different folding mechanisms, and (iii) the reconstruction of a phylogenetic tree using alignment and Profile Neighbor Joining (PNJ) treeing programs that process sequence and structure information simultaneously.

## Materials and Methods

### Sequence annotation

A set of 1287 unique and mostly unclassified Neocallimastigomycota rRNA gene sequences was retrieved from the GenBank NT (Nucleotide) database [30]. For an exact delineation and precise identification of the ITS1 region, an annotation step was introduced similar to that of Keller *et al.* [31]. This enabled the identification of regions bordering ITS1 using Hidden Markov Models, which were generated as follows. All sequences matching the search terms “(18S rrna[Gene Name]) AND Fungi[Organism]” (18912) for 18S and “(5.8S rrna[Gene Name]) AND Fungi[Organism]” (15468) for 5.8S were obtained from GenBank (date 23.05.2012). To avoid a bias towards more frequently sequenced species, only one representative out of each species-group was chosen randomly. Based on the reduced datasets, 3327 18S rRNA and 5232 5.8S rRNA gene sequences were aligned using ClustalW2 [32], and manually refined by eliminating 79 18S and 136 5.8S sequences that were too short or obviously wrongly aligned. The boundaries of the ITS1 region were identified by comparison with high quality rRNA gene sequence alignments from the SILVA database [33] and ITS1 sequence annotations by Tuckwell *et al.* [34]. The last 25 nucleotides (nt) at the 3'-end of the 18S rRNA gene and the first 25 nt at the 5'-end of the 5.8S rRNA gene were then extracted. Profile Hidden Markov Models based on both extracted 25 nt alignments were created using the HMMER suite 2.3.2 [35]. By applying both fungi HMMs to the neocallimastigomycete sequences, 604 18S rRNA and 776 5.8S rRNA gene regions could be identified. These were used to create four Neocallimastigomycota-specific HMMs consisting of 25 and 10 nucleotides for 18S and 5.8S, respectively. HMMs containing 25 nucleotides were disregarded, as many sequences available in NCBI do not include overlaps with or extend far enough into the 18S or 5.8S rRNA genes. The shorter HMMs were applied for the final sequence annotation with an e-value of 0.1. An overview of this process including secondary structure prediction is given in Figure S1.

### Secondary structure prediction and motif detection

**Figure 1:**
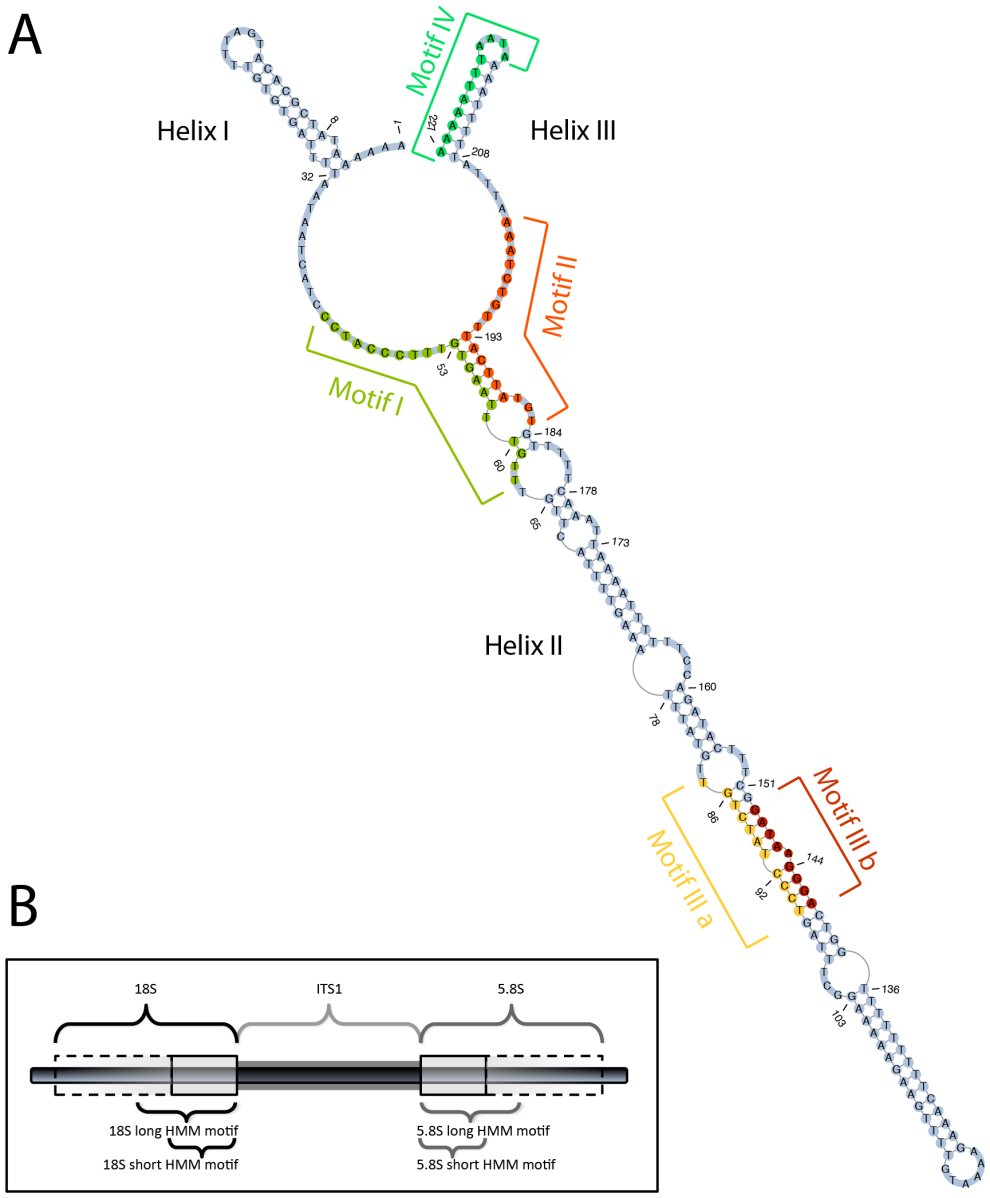
Visualization of secondary structure, sequence motifs and sequence annotation. (**A**) The secondary structure of the ITS1 region of JF423742 (selected as an example) illustrates the common core shape observed in all analysed neocallimastigomycete sequences. It folds into a three helix conformation, with two short helices (I and III) and a longer one in between (II). Additionally, four very conserved sequence motifs are highlighted in the structure. (**B**) The black bar shows a nucleotide string of rDNA. On the left is the terminus of the 18S rRNA gene, with its 18S HMM. Together with the 5.8S HMM motif on the right, both enable an exact delineation of the ITS1 region.

Sequences were folded (i) by energy minimization using UNAFold 3.8.1 [36] or (ii) by a helix-wise divide and conquer approach. The latter method makes use of prior knowledge of sequence motifs to divide a sequence into several parts, according to the presumed location of the helices. Each part was folded separately using UNAFold and concatenated afterwards to build the full structure. The necessary sequence motifs were predicted by expectation maximization on the annotated dataset using MEME Suite 4.8.1 [37]. Only motifs identified in all annotated sequences were considered. Two motifs (Figure 1 motifs I and II), close to the start and end of the second helix were extracted from MEME to build motif specific Hidden Markov Models. Those were used to identify the inter helix regions with an e-value cut-off of 0.001.

### Sequence-structure alignment and tree reconstruction

A global multiple sequence-structure alignment (File S1) was automatically generated in 4SALE v1.7 [16], [17], whereby sequences and their individual secondary structures were synchronously aligned using a sequence-structure specific scoring matrix [16]. 4SALE uses ClustalW [32] but with a specified scoring matrix, fitted to a 12-letter alphabet specifically constructed for sequence-structure data. Hence 4SALE does not use a 4×4 scoring matrix but rather a 12×12 matrix for each nucleotide, with its three structural states (paired left, paired right, or unpaired). Based on the simultaneous consideration of the primary sequence and secondary structure information, phylogenetic relationships were reconstructed by (Profile-)Neighbour-Joining through the use of a sequence€structure specific general time reversible (GTR) substitution model as implemented in ProfDistS v0.9.9 [18], [38], [39]. The tree was finally visualized using FigTree 1.3.1 [40].

### Compensatory base changes

Compensatory base changes (CBCs) were analyzed and graphics exported using 4SALE. In the case of the helix-wise analysis, a truncated alignment was loaded into 4SALE to count CBCs in a helix specific manner.

## Results

### Sequence annotation

The annotation of 1287 unique Genbank sequences using the long (25 nt) Neocallimastigomycota-specific Hidden Markov Models and an e-value of 0.001 resulted in 1032 hits for the 18S rRNA gene, and 793 hits for the 5.8S rRNA gene motif. 554 sequences (297 of which were unique based on the ITS1 sequence alone) contained both motifs and provided a full annotation of the ITS1 region. Applying the shorter (10 nt) HMMs on the same dataset required a lower e-value which was set to 0.1. Although the risk of false annotations increases in this case, 1120 ITS1 sequences (606 of which were unique; 1150 hits for the 18S and 1244 hits for the 5.8S region) could be annotated using this method.

### Prediction and description of a common core secondary structure

All annotated and unique ITS1 sequences were folded by energy minimization and the number of helices was counted (see Table 1). Of the 606 structures obtained, 476 sequences (78.55 per cent) folded into a three helix structure.

**Table 1:** Secondary structures of anaerobic fungal ITS1 sequences.

Helices	2	3	4	5	6	7	8	9
Structures	2	**476**	80	38	3	2	4	1
Percentage	0.33	**78.55**	13.2	6.27	0.5	0.33	0.66	0.17

Number and per cent of anaerobic fungal ITS1 secondary structures that folded into a total of 2 to 9 different helices using energy minimization by UNAFold 3.8.1.

The three-helix structures were inspected manually for shapes and helix lengths. 381 of the 476 structures (80.04 per cent) contained a common core shape. A representative structure is shown in Figure 1. This core consists of two short helices (helix I, 12 to 35 nt long; helix III, 8 to 23 nt long) near the 5' and 3' termini, and a long helix (helix II, 115 to 201 nt long) in between. All three helices are located around a central ring separating helix I from helix II and helix II from helix III, with 15 to 28 and 10 to 29 unpaired nucleotides in the inter-helix regions, respectively. Both inter-helix regions are very conserved. This high conservation is supported by a motif analysis which revealed two long sequence motifs (Figure 1, motifs I and II) starting at the beginning and at the end of helix II.

Two additional motifs were detected among all sequences, confirming the motif analysis of Tuckwell *et al.* [34]. A complementary motif is located towards the second half of helix II (Figure 1, motifs IIIa and IIIb), and a further motif, motif IV, is located at the closing stem of helix III.

**Figure 2:**
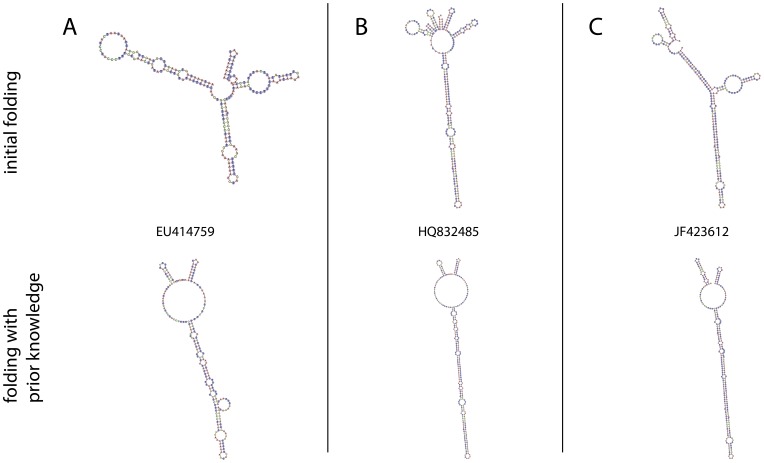
Secondary structures of the ITS1 region from three different sequence types. ITS1 secondary structures from (**A**) EU414759, (**B**) HQ832485 and (**C**) JF423612. The illustrated structures did not fold into the typical three helix conformation when folded initially with UNAFold (upper images). By breaking the sequences into three parts based on the known location of sequence motifs, each part could be folded individually. The concatenated helices (bottom images) result in the typical three-helix common core structure predicted by the direct folding of the majority of all annotated sequences.

**Figure 3:**
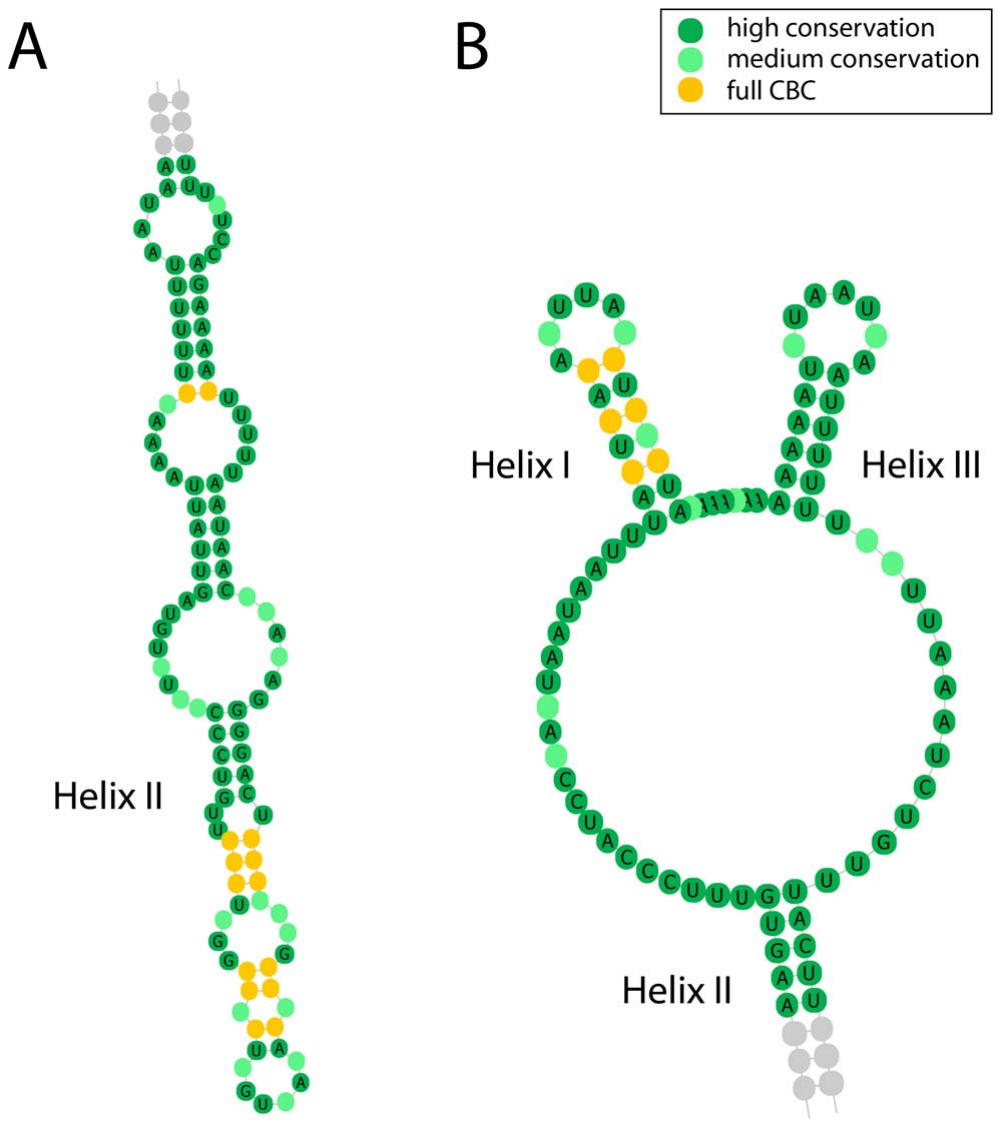
CBC visualization of neocallimastigomycete ITS1 regions. (**A**) Parts of the consensus secondary ITS1 structure of GQ850303 and JF423532 from helix II. Marked in yellow are compensatory base changes between the two sequences. (**B**) Partial consensus secondary ITS1 structure of JF423714 and JX184822. CBCs occurring in helix I are highlighted in yellow.

In contrast to the 381 sequences that folded into this common core structure, the 225 remaining structures (Table 1) did not automatically fold into the common shape using energy minimization. However, a specific helix-wise folding approach further improved folding quality and supported the proposed three-helix structure. Sequence motifs close to the second helix were identified and used to separate each of these 225 sequences into three proposed helix domains. By folding each of the three proposed helix domains individually, and then concatenating the structures, the number of sequences that folded into the typical three helix conformation increased to 575 of 606 (94.88 per cent). Examples of three sequences (EU414759, HQ832485 and JF423612) that did not fold correctly using the initial whole sequence folding method are illustrated in Figure 2. The upper images show the ITS1 secondary structures when the sequences were folded as a whole. In comparison, the images at the bottom show the same sequences but with the secondary structure that was obtained by folding each of the three proposed helix domains separately before concatenating the three structural elements. These structure analyses revealed several CBCs, which mainly occurred in the first and second helix of the common core. As an example, parts of two CBC-rich consensus structures are depicted in Figure 3. The consensus between sequences GQ850303 and JF423532 shows six of seven full CBCs towards the end of helix II close to the loop region (Figure 3A). Sequences JF423714 and JX184822 shared several CBCs in helix I (Figure 3B). Only very few CBCs were observed in helix III. Based on the structural elements of folded sequences, an adenine + thymine (AT) content analysis was performed (Table 2). In general, a high AT content (compared to other eukaryotes) of 79 per cent was found for the analyzed neocallimastigomycete ITS1 sequences.

**Table 2:** AT content of different structural elements from neocallimastigomycete ITS1 regions in per cent.

Start	Helix I	Inter I	Helix II	Inter II	Helix III	Stop	ITS1
0.95	0.63	0.70	0.77	0.84	1	1	0.79

The “Start” and “Stop” region marked in the Table represent the nucleotides before the first and after the third helix, respectively. “Inter 1” and “Inter 2” represent interhelical regions between first and second and second and third helix, respectively.

A web page for the annotation and secondary structure prediction of neocallimastigomycete sequences is available online at: https://www.anaerobicfungi.biocommons.org.nz.

### Tree reconstruction

**Figure 4:**
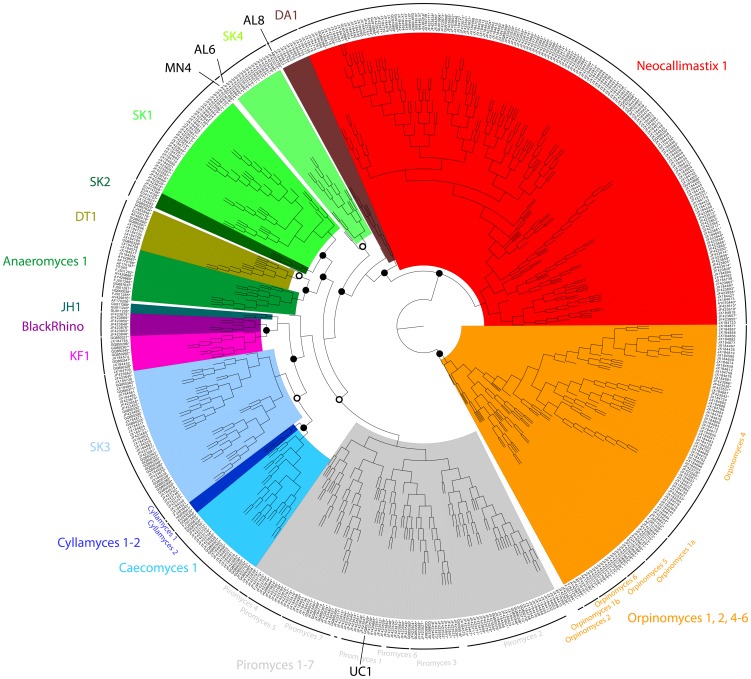
Profile Neighbor Joining tree of neocallimastigomycete ITS1 sequences. Profile Neighbor Joining tree calculated using sequence and structure data from 1120 (575 unique) complete neocallimastigomycete ITS1 sequences with 1000 bootstrap replicates in 29 iterations. Open circles indicate bootstrap values in a range of 50 to 90, closed circles indicate a bootstrap value above 90, and the scale bar indicates the distance. The tree contains the six known genera *Anaeromyces*, *Caecomyces*, *Cyllamyces*, *Neocallimastix*, *Orpinomyces* and *Piromyces*. and the unclassified sequences GQ850325, AF170206 and AF170205. Other monophyletic groups are highlighted and named according to Kittelmann *et al.* [10]. A total of ten sequences did not cluster into any of the defined groups and were named according to their accession numbers only (JF423517, JF423484, JF423882, GU055516, JX184570, JF423626, JF423625, GQ850325, AF170205, and AF170206). Sequences, accession numbers and taxonomic classifications (including definitions of subclusters of the genera *Cyllamyces*, *Orpinomyces* and *Piromyces*) are available from the taxonomy file (File S2). Subcluster Orpinomyces 3 is not represented in this tree due to the lack of full-length ITS1 sequences for this group. A comparison of bootstrap values from this study to the sequence–only analysis of Kittelmann *et al.* [10] is given in Table S1. YE505  =  *Anaeromyces mucronatus* YE505.

The 575 aligned neocallimastigomycete sequence-structures yielded a Profile Neighbor Joining tree that resolved the six known genera *Anaeromyces*, *Caecomyces*, *Cyllamyces Neocallimastix*, *Orpinomyces* and *Piromyces* as distinct monophyletic groups in agreement with the analyses of Kittelmann *et al.* (2012). Accession numbers of sequences used in both trees are marked with an asterisk in Figure 4. The taxonomic assignments differed for only 20 out of a total of 348 unique sequences between the sequence-only phylogeny [10] and the sequence-structure-based phylogeny we present here. Fourteen of these differences were due to our decision to split up the group formerly known as “Orpinomyces 1” into two clusters, namely “Orpinomyces 1a” and “Orpinomyces 1b” to acknowledge that this group does not cluster monophyletically when using both sequence and structure information for treeing. Additionally, three sequences formerly clustering into “Neocallimastix 1” clustered into the genus *Orpinomyces* when using both sequence and structure information (AF170205, JF423625, and JF423626). They could therefore not clearly be assigned to a genus and are listed with accession numbers only (File S2). Three further sequences clustered consistently into the genus *Piromyces* but clustered into different subgroups depending on the method used (JF423517, JF423484 and JF423882). Therefore, in the revised taxonomy (this study) these three sequences are only classified to the genus level. At a higher level, the genus *Orpinomyces* forms a sister group to the genus *Neocallimastix* and to the radiation of *Piromyces*, *Cyllamyces*, *Caecomyces* and *Anaeromyces*. In this later group, the sister groups *Cyllamyces* and *Caecomyces* cluster together with *Piromyces* and distinct from *Anaeromyces*. This is in contrast to the analysis of Kittelmann *et al.* (2012), where the sister groups of *Cyllamyces* and *Caecomyces* formed a cluster separate from the assemblage of *Piromyces* and *Anaeromyces*, albeit with low bootstrap support.

Beside the six known genera of Neocallimastigomycota, several novel groups as identified in previous studies [8]–[11], [41] could be confirmed: AL6, AL8, BlackRhino, DT1, JH1, KF1, MN4, SK1, SK2, SK3, SK4 and UC1 form monophyletic clusters within the tree. In addition, we detected a further novel monophyletic cluster, named DA1, which contains sequences derived from rumen content and faeces of pasture-fed lactating dairy cows in New-Zealand (Al-Halbouni & Jarvis, unpublished data). However, it remains unknown whether some of these novel groups represent new genera or species. In agreement with Kittelmann *et al.* (2012), SK1 forms a sister group to SK2, both clustering closely to DT1. SK3, previously clustering near *Orpinomyces*, now is sister group to the *Caecomyces* and *Cyllamyces* clades. In both phylogenies, KF1 and BlackRhino form sister groups closely related to JH1; UC1 typically clusters within the genus *Piromyces*.

## Discussion

To our knowledge, this is the first proposed common secondary structure model of the ITS1 of the Neocallimastigomycota. The ITS1 is not conserved within the Eukarya, and so not unexpectedly it differs from ITS1 secondary structure models found for other groups within the Eukarya [25]–[28]. Our proposed conformation for the Neocallimastigomycota consists of a small ring with three helices, the second one typically being the longest. Although analysis of the AT content shows that the majority of base pairings consist of the less stable AT-bonds forming only two hydrogen connections (three for the more stable GC-bonds), the structure seems to be highly conserved, especially in the first and second helix.

This is supported by the large number of initial structures – 381 out of 606 sequences – that folded precisely into the proposed common core shape. No other similar common pattern could be identified among the remaining foldings. A total of 225 sequences did not fold immediately into the three helix shape. This might be due to the high AT content in sequences in members of this phylum. We assume that a reduced selection of those nucleotides results in an increased number of possible pairing conformations together with a higher probability of incorrect folding. Therefore, incorrect foldings might also result in good energy values and a high number of closed base pairs. These sequences, however, belonged to a broad range of anaerobic fungal groups, suggesting that the different folding was not specific to particular taxonomic groups within the phylum (Table 3).

**Table 3:** Number of sequence-structure combinations included in phylogenetic tree reconstruction, ordered by taxonomic group.

Taxonomic group	Direct and helix-wise folding	Helix-wise folding only	Total
AF170205	1	-	1
AF170206	-	1	1
AL6	-	1	1
AL8	-	1	1
Anaeromyces 1	12	4	16
BlackRhino	5	2	7
Caecomyces 1	24	1	25
Cyllamyces 1	1	-	1
Cyllamyces 2	3	-	3
DA1	7	2	9
DT1	6	7	13
GQ850325	-	1	1
GU055516	1	-	1
JF423484	1	-	1
JF423517	1	-	1
JF423625	1	-	1
JF423626	-	1	1
JF423882	-	1	1
JH1	-	3	3
JX184570	1	-	1
KF1	7	4	11
MN4	1	-	1
Neocallimastix 1	127	53	180
Orpinomyces 1a	2	7	9
Orpinomyces 1b	4	1	5
Orpinomyces 2	2	-	2
Orpinomyces 4	28	38	66
Orpinomyces 5	3	4	7
Orpinomyces 6	5	5	10
Piromyces 1	11	-	11
Piromyces 2	27	3	30
Piromyces 3	10	4	14
Piromyces 4	3	-	3
Piromyces 5	12	-	12
Piromyces 6	4	3	7
Piromyces 7	14	1	15
SK1	10	26	36
SK2	-	5	5
SK3	39	6	45
SK4	5	11	16
UC1	-	1	1

Sum	378	197	575

Sequences could either be folded into the three-helix core structure using both direct folding and helix-wise folding (direct and helix-wise folding) or only by helix-wise folding (helix-wise folding only). All 575 sequence-structure combinations were used for phylogenetic tree inference.

The central ring contains several unpaired nucleotides that are likely to interact with parts of the first or second helix during *in silico* folding routines. This too would result in incorrect foldings. However, these difficulties can be addressed by combining prior knowledge of sequence conservation with a “divide and conquer” folding technique. The helix-wise folding method we applied guided the folding process and significantly reduced the number of different foldings. It confirmed the identified core structure in 94.88 per cent of sequences (575 out of 606 folds). Some of the remaining 5.12 per cent show a very similar conformation to the proposed structure or parts of it, but contain larger bulbs or a Y-shaped helix. This might again be due to the high AT content, which may cause folding predictions that differ from the consensus model. In this case, an individual Homology Modelling folding approach [42] may help revealing the shape of those sequences, although it would cause a mixture of different folding methods. Benefits and pitfalls of Homology Modelling have been discussed by Markert *et al.* [43]. Some other sequences within the 5.12 per cent were probably wrongly annotated and differed strongly in both sequence length and folding conformation. This might be due to the use of short HMMs (10 nt) to identify the flanking 18S and 5.8S rRNA gene regions in combination with a low e-value of 0.1. The use of short HMMs was necessary because many sequences available in NCBI do not include overlaps with or extend far enough into the 18S or 5.8S rRNA genes. Thus, HMM hits are less significant, and require a lower e-value cut-off. Even so, the number of wrongly-annotated sequences makes up only a small proportion of the 5.12 per cent of differently folded sequences.

The identification of CBCs in the first and second helix of ITS1 from different genera supports the high degree of conservation of the proposed core structure. While helix I is well supported by CBCs throughout, helix II is supported by CBCs close to the loop region. However, no CBCs were observed in helix III, which might be due to the very conserved sequence motif that covers the terminal stem of that helix, allowing little change in those nucleotides and limiting the potential for CBCs. The complementary stem of helix III shows more variation, which is reflected, e.g., by small bulges containing free nucleotides. These were especially observed in sequences belonging to the SK4 group. Motif analysis revealed several very conserved sequence motifs throughout all the sequences analyzed.

Although the Neocallimastigomycota comprise a whole phylum, which at present is represented by only one family, it covers less sequence variation than can be found in other phyla. By combining HMM sequence annotation and knowledge of conserved motifs, together with a divide and conquer folding approach, we were able to identify a common core structure, which allows us to perform a full sequence-structure analysis based on the phylogenetic pipeline described by Schultz and Wolf [29]. The calculated PNJ tree resolves all of the six known genera as monophyletic groups (Figure 4). The *Cyllamyces* clade, however, clusters as a monophyletic alliance within the *Caecomyces* genus in the distance tree which does not make use of PNJ iterations (Figure S2). When iterating the PNJ algorithm [38], however, both genera are reliably resolved as sister groups. Regarding the remaining genera, only three sequences which previously were categorized as Neocallimastix 1 now reside within the *Orpinomyces* clade, but branch closely to the basal split of both groups and show a weak bootstrap support. This and small within-genus differences in the clustering of groups when compared to the earlier phylogeny of Neocallimastigomycota [10] might be due to several reasons. In the current study, phylogenetic analysis of Neocallimastigomycota sequences was performed on sequence and secondary structure simultaneously for the first time. Keller *et al.* [24] showed that a combined sequence and structure analysis improves the accuracy and the robustness of trees on higher phylogenetic levels. Other reasons might be the extended taxon sampling or the different treeing method applied for the sequence-structure-based tree calculation. In total, 575 unique of 1120 annotated sequences were included into the tree reconstruction. This number is approximately 1.4 times that used by Kittelmann *et al.* [24]. Finally, our study included all sequences, available from public databases, which were automatically processed without any manual interventions. This results in unbalanced taxon sampling and underrepresented groups. For example only one to three sequences represent the groups of AL6, AL8, JH1, UC1 and MN4 and groups AL1-AL5, AL7, MN3 and Orpinomyces 3 were not included as no full-length sequences are available for these. As more full-length ITS1 sequences become available, these might in future form more stable groups within the tree.

A high percentage of sequences (79 per cent) folded into the proposed 3-helix common core structure *in silico* without coaching, indicating that our proposed secondary structure likely represents the actual structure of anaerobic fungal ITS1 sequences. The structures were not verified by *in vitro* analyses, but computationally predicted ITS2 secondary structures, with similar helix lengths, significantly improve robustness and accuracy of reconstructed phylogenies (Keller *et al.* [24]). However, even though the phylogenies are more robust and accurate when using such computationally predicted secondary structures, dimethyl sulfate analyses of anaerobic fungal species may additionally help to further improve ITS1 sequence-structure alignments in the future. The proposed secondary structure allowed us to automate the alignment and calculate a robust phylogenetic tree, which, in contrast to earlier studies, is not based on primary sequence information alone, but gains additional stability through the inclusion of secondary structure information. Our approach provides reproducible alignments, thus allowing the exact assessment of base differences of novel environmental sequences to describe type species and placing potential new lineages within the existing scheme. This is useful (1) for community diversity descriptions based on large amounts of data, and (2) for identifying novel candidate taxa that might warrant further effort for complete description. According to article 32 of the Vienna Code [44], the description of new taxa based on pure sequence information must include specific references to the molecular characters that distinguish the taxon (e.g., specific differences in nucleotide positions in a molecular alignment; [45], [46]). The implementation of using both primary sequence and secondary structure information for the taxonomic classification of anaerobic fungi could represent a first step towards developing broadly accepted standards for sequence-based taxonomy where isolated species are still lacking [12]. The taxonomic framework we present is compatible with software such as QIIME [47] frequently used for the taxonomic assignment of reads from next-generation sequencing technologies. Once detected, novel anaerobic fungal ITS1 sequence types from isolates or environmental samples can be easily annotated, folded, aligned and added to the established taxonomic framework. In the future, expansion of this framework especially through an effort of collating sequence information of validly described isolates will help to continuously improve anaerobic fungal community structure analysis based on the ITS1 gene. The secondary structure conformation could not be transferred to further fungal groups like yeasts in a first attempt, but the methodologies we applied here have potential to refine ITS1 sequence annotation as well as to improve secondary structure prediction and phylogenetic reconstructions of other fungal groups. A combination of structures from *in vitro* analysis and energy-based folding (see Lamanna *et al.* [48]) could further improve secondary structure prediction, especially in ambiguous cases.

Supporting data are available online: File S1–S2 and Figures S1–S2.

## Supporting Information

Figure S1 **Flow chart of HMM generation, sequence annotation and secondary structure prediction.** Flow chart describing the HMM generation process used for the ITS1 sequence annotation of Neocallimastigomycota on the left and sequence annotation with secondary structure prediction on the right. Blue cylinders represent data retrieved from NCBI, green diamonds represent decisions, dark blue boxes represent actions and preliminary results, and orange or red cylinders represent intermediate or final data sets, respectively.
(TIF)

Figure S2 **Distance tree of neocallimastigomycete ITS1 sequences.** Distance tree calculated using sequence and secondary structure data from 575 complete neocallimastigomycete ITS1 sequences with 1000 bootstrap replicates (only values between highlighted groups and above 50 are shown). Open circles indicate bootstrap values in a range of 50 to 90, closed circles – a bootstrap value above 90, and the scale bar represents the evolutionary distance. The tree contains the six known genera *Anaeromyces*, *Caecomyces*, *Cyllamyces*, *Neocallimastix*, *Orpinomyces* and *Piromyces*. Other monophyletic groups are highlighted and named according to Kittelmann et al. [10]. Sequences, accession numbers and taxonomic classifications (including definitions of subclusters of the genera *Cyllamyces*, *Orpinomyces* and *Piromyces*) are available from the taxonomy file (File S2). Subcluster Orpinomyces 3 is not represented in this tree due to the lack of full-length ITS1 sequences for this group. A comparison of bootstrap values from this study to the sequence–only analysis of Kittelmann *et al.* [10] is given in Table S1. YE505 =  *Anaeromyces mucronatus* YE505.
(TIF)

File S1 **Sequence-structure alignment (.xfasta format) containing all 575 sequences and structures used for phylogenetic tree reconstruction.**
(XFASTA)

File S2 **Accession numbers, taxonomic classifications and sequence data of all sequences used in this study.** The sheet named “DatabaseForTaxonomicAssignment” contains the representatives of 575 unique full-length sequences used for tree construction and corresponding secondary structures obtained by direct or helix-wise folding (see table header {1}) and 89 partial sequences that could be defined at the 5'-end using primer MN100F (31) and at the 3'-end using the developed HMM (see table header {2}). This database comprises 664 sequences that can be converted into a fasta sequence file (containing accession numbers and sequence information) and a txt taxonomy file (containing accession numbers and taxonomic information) and used for the taxonomic assignment of environmental sequencing reads collected using high-throughput next-generation sequencing technologies. The sheet named “ExcludedSequences” contains 95 full-length or partial sequences that should not be used for taxonomic assignment of anaerobic fungi without further detailed inspection for one of the following reasons: 1. sequences showed foldings different to the proposed 3-helix common core structure (see table header {3}), 2. sequences were incomplete at the 5'-end and the second and third helices could not be defined based on primer MN100F (see table header {4}), 3. sequences were incomplete at the 5'-end and were previously assigned to Orpinomyces 1 (see table header {5}), or 4. sequences were incomplete at the 3'-end (see table header {6}). Finally, the sheet named “ComparisonToKittelmannEtAl2012” contains a comparison of taxonomic assignments based on sequence and structure information (this study) with those derived from a sequence-only phylogeny (Kittelmann *et al.* [10]). The highlighted rows indicate sequences for which taxonomic classification differed between the two studies. A detailed information for these differences is provided in the “Comment” column.
(XLS)

Table S1 **Comparison of bootstrap replicates of this study to the sequence–only analysis by Kittelmann** ***et al.*** **** [10] **.**
(XLSX)
