# Supplementary figures and images for: Internal Transcribed Spacer 1 Secondary Structure Analysis Reveals a Common Core throughout the Anaerobic Fungi (Neocallimastigomycota)
Source: PLoS One. 2014 Mar 24;9(3):e91928. doi: 10.1371/journal.pone.0091928 (PMC3963862; doi:10.1371/journal.pone.0091928)

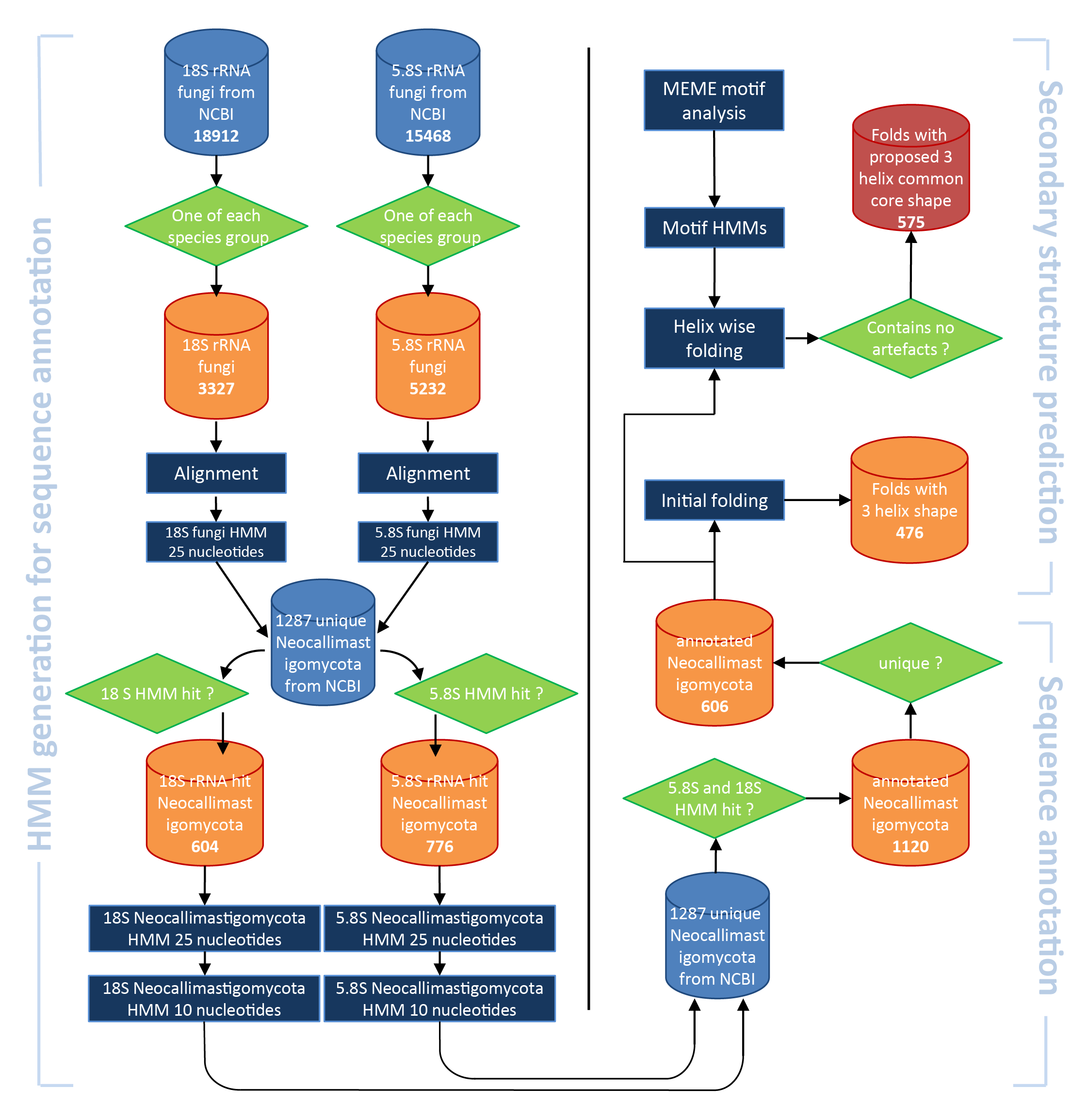

Supplement: Figure S1 — Flow chart of HMM generation, sequence annotation and secondary structure prediction. Flow chart describing the HMM generation process used for the ITS1 sequence annotation of Neocallimastigomycota on the left and sequence annotation with secondary structure prediction on the right. Blue cylinders represent data retrieved from NCBI, green diamonds represent decisions, dark blue boxes represent actions and preliminary results, and orange or red cylinders represent intermediate or final data sets, respectively. (TIF) [file pone.0091928.s001.tif]
